# Breaking a Dogma: High‐Throughput Live‐Cell Imaging in Real‐Time with Hoechst 33342

**DOI:** 10.1002/adhm.202300230

**Published:** 2023-03-31

**Authors:** Heiko Fuchs, Kirsten Jahn, Xiaonan Hu, Roland Meister, Maximilian Binter, Carsten Framme

**Affiliations:** ^1^ Institute of Ophthalmology University Eye Hospital Hannover Medical School Carl‐Neuberg Strasse 1 30625 Hannover Germany; ^2^ Department of Psychiatry Social Psychiatry and Psychotherapy Hannover Medical School Carl‐Neuberg Strasse 1 30625 Hannover Germany

**Keywords:** fluorescent microscopy, high‐throughput analysis, Hoechst 33342, real‐time live‐cell imaging

## Abstract

Automated high‐throughput live cell imaging (LCI) enables investigation of substance effects on cells in vitro. Usually, cell number is analyzed by phase‐contrast imaging, which is reliable only for a few cell types. Therefore, an accurate cell counting method, such as staining the nuclei with Hoechst 33342 before LCI, will be desirable. However, since the mid‐1980s, the dogma exists that Hoechst can only be used for endpoint analyses because of its cytotoxic properties and the potentially phototoxic effects of the excitation light. Since microscopic camera sensitivity has significantly improved, this study investigates whether this dogma is still justified. Therefore, exposure parameters are optimized using a 4× objective, and the minimum required Hoechst concentration is evaluated, allowing LCI at 30‐min intervals over 5 days. Remarkably, a Hoechst concentration of only 57 × 10^−9^ m significantly inhibits proliferation and thus impairs cell viability. However, Hoechst concentrations between 7 × 10^−9^ and 28 × 10^−9^ m can be determined, which are neither cytotoxic nor impacting cell viability, proliferation, or signaling pathways. The method can be adapted to regular inverted fluorescence microscopes and allows, for example, to determine the cytotoxicity of a substance or the transduction efficiency, with the advantage that the analysis can be repeated at any desired time point.

## Graphical abstract

By adding Hoechst 33342 to the media before live cell imaging (LCI), the method presented here permits accurate cell counting in various adherent cells, including primary cells and those not reliably detected in the automated cell count due to their morphology. The method enables, for example, real‐time analysis of proliferation kinetics, cytotoxicity of a chemical, or transduction efficiency at any time.

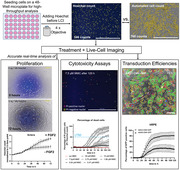

## 1 Introduction

Live cell imaging (LCI) analyses are becoming increasingly crucial in biotechnology and medicine. High‐throughput analyses such as automated LCI procedures are performed to investigate the effect of novel compounds or drugs in vitro. In personalized medicine, high‐throughput studies using automated LCI techniques enable in vitro testing of primary patient cultures following specially formulated medications for each patient. Multiwells, such as 48‐ or 96‐well plates, are utilized for high‐throughput LCI investigations to evaluate many conditions and replicates simultaneously.^[^ ^1^ ^]^ However, with smaller diameters of the wells, phase contrast images are compromised by the so‐called “meniscus effect,” resulting that adhesion forces causing a concavely curved liquid surface. As a result, phase contrast images are significantly overexposed toward the edges, and the cells in this area stand out poorly from the background, making it impossible to identify them.

Consequently, an image preprocessing step that involves rolling ball background subtraction must be completed before cells can be counted using analytic software. Since a new image must be created for each phase contrast image, this image processing step is time‐consuming and requires additional data storage. Object gating can be utilized to determine the number of cells after processing. For this purpose, the minimum and maximum object sizes and background subtraction must be defined for gating. This cell counting approach performs pretty accurately, even though only with cells that are visibly distinct from surrounding cells in the phase contrast image and have comparable cell size, length, and width. In any case, preprocessing and cell gating settings such as minimum and maximum size parameters and rolling ball subtractions are user‐dependent, resulting in partly unobjective, user‐dependent counts.

Since this counting method did not work for the cell types we were interested in, including primary human sclera fibroblast, we looked for alternate methods to count the cells more precisely.

Another method for counting cells is to use fluorescent dyes. The cell nuclei and hence the number of cells are visualized, for instance, using the bisbenzamide derivative Hoechst. The advantage of counting cells via Hoechst staining is that the nucleus size often does not vary as much as the cell size. In addition, in confluent cells, where automated cell counting often fails, the nuclei are usually far enough apart to be gated. Furthermore, the meniscus effect does not affect fluorescence imaging acquisition, and the dyed cell nuclei can be easily distinguished from the background, making additional image preprocessing superfluous. One of the first Hoechst derivatives developed, Hoechst 33258, was already used in 1969 for nuclear staining in living animals^[^ ^2^ ^]^ of cell culture^[^ ^3^ ^]^ and for karyotyping.^[^ ^4^ ^]^ In 1978, another DNA‐binding fluorochrome, Hoechst 33342, was reported as a live stain of cells suitable for subsequent cell sorting.^[^ ^5^ ^]^ Comparing the two DNA‐binding fluorochromes revealed that Hoechst 33342 is better suited as a live stain for various cell types than Hoechst 33258.^[^ ^6^ ^]^

However, sobering soon set in since numerous studies claimed that Hoechst significantly disrupts cell cycle balance, suppresses proliferation, and reduces cell viability.^[^ ^7^ ^]^ Hoechst is reported to disrupt DNA replication and RNA synthesis because it binds to AT‐rich DNA regions.^[^ ^8^ ^]^ Another study revealed that Hoechst impairs neural growth factor (NGF) induced neuronal development, myofibroblast differentiation,^[^ ^9^ ^]^ and embryonic carcinoma differentiation.^[^ ^10^ ^]^ Different studies demonstrated that Hoechst‐exposed cells would have reduced vitality and behavior, making them unsuitable for real‐time LCI analysis.^[^ ^11^ ^]^ Hoechst's excitation wavelength range, which has an excitation peak at 365 nm and partially overlaps with the UV‐A wavelength range (315–400 nm), is another issue since it may have phototoxic impacts.^[^ ^11^ ^]^

All these findings from the eighties resulted in the dogma that Hoechst cannot be used during LCI. Therefore, Hoechst has been used for LCI analysis mainly as an endpoint analysis step.

In the above publications, 1 × 10^−6^ to 20 × 10^−6^ m Hoechst was typically employed for live staining. One goal of this study was to determine whether the previously utilized Hoechst concentrations from the eighties are still required with today's constantly advanced technology in microscope cameras. To enable long‐term high‐throughput LCI analysis with Hoechst, (i) a 4× objective was used to analyze as many cells as possible per image section, (ii) exposure parameters were optimized to avoid potential phototoxic effects, and (iii) an appropriate Hoechst concentration was determined that allows LCI at 30‐min intervals for up to 5 days without affecting proliferation rates, viability, or physiological behavior.

## 2 Results

**Figure 1:**
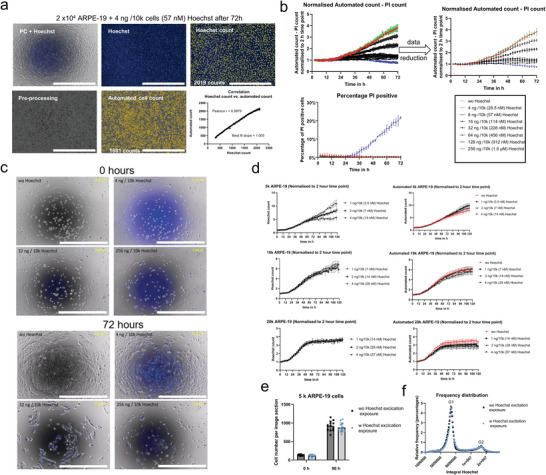
a) Comparison of automated cell count and Hoechst cell count on 2 × 10^4^ ARPE‐19 cells exposed to 4 ng/10^4^ cells (57 × 10^−9^ m) Hoechst for 72 h. Image preprocessing was performed for the automated count, and an object size between 15 and 70 µm was used for cell gating. For the Hoechst count, Hoechst‐stained (blue) nuclei were gated using object sizes between 7 and 30 µm. With 4 ng/10^4^ cells Hoechst, both counting methods showed similar cell numbers after 72 h. b) Testing different Hoechst concentrations, ranging from 256 ng/10^4^ cells down to 4 ng/10^4^ cells on 1 × 10^4^ ARPE‐19 cells in 20 min intervals for 72 h. Propidium iodide (PI) was additionally added to estimate the number of alive cells (upper two subfigures) and the percentage of dead cells (lower subfigure). A data reduction step was carried out for a more transparent presentation (every fifth data point was plotted). c) Representative phase‐contrast combined with Hoechst images of ARPE‐19 cells exposed to none, 4 ng/10^4^ cells, 32 ng/10^4^ cells, and 256 ng/10^4^ cells at the beginning of LCI and 72 h later. PI‐positive nuclei highlighted in red reveal dead cells. The scale bar represents 1000 µm. d) Testing minimum Hoechst concentrations on ARPE‐19 cells in combination with different cell densities. LCI was performed for 120 h, and four technical replicates were evaluated. A total of 5 × 10^3^ (upper two subfigures), 1 × 10^4^ (middle two subfigures), and 2 × 10^4^ (lower two subfigures) ARPE‐19 cells were seeded and exposed to none, 1, 2, or 4 ng/10^4^ cells Hoechst. The Hoechst count (normalized to the cell count after 2 h LCI) is shown on the left. The automated count (normalized to the cell count after 2 h LCI) is presented on the right, and the non‐Hoechst exposed cells are highlighted in red. e) Cell number of 5000 seeded ARPE‐19 cells per image section at 0 and 96 h after LCI. ARPE‐19 cells were either not exposed (black squares) or exposed for 71 ms in 20 min intervals for 96 h (blue dots) with Hoechst excitation light. The data were collected from 12 technical replicates each. f) The frequency distribution of the integral (Hoechst intensity per area) of 12 technical replicates from an average of 75 000 nuclei was determined from ARPE‐19 cells that were not irradiated (black squares) or were irradiated as described in Figure 1d (blue dots).

First, we compared the accuracy of automated versus Hoechst cell counting and stained primary human tenon, sclera, retinal pigment epithelial (RPE) cells, and the neuroblastoma cell line SH‐SY5Y with 1 × 10−6 M Hoechst (Figure S1, Supporting Information). We observed significant discrepancies between these two counting methods depending on the cell type, up to 100% in some cases. It is apparent here that accurate cell counting is impossible with automated phase‐contrast cell counting if, for example, the cells are pigmented or have a variable cell shape and size. Therefore, we investigated the possibility of using Hoechst not only for LCI endpoint analyses but also during an LCI experiment. For better comparability, we chose the RPE cell line ARPE‐19 because it can be adequately counted with the automated cell count due to its morphology and nearly uniform cell size. The correlation plot revealed nearly identical count results between the automated and the Hoechst count after 72 h from four technical replicates. Pearson's *r* was 0.9979 with a Best fit slope of 1.005 (**Figure** **1**a).

In an initial screening, we tested different concentrations between 256 and 4 ng Hoechst per 10 000 cells, corresponding to molarity between 1.8 × 10^−6^ and 28.5 × 10^−9^ m at a molecular weight of 562 g L^−1^ for Hoechst. LCI was performed in 20‐min intervals for 72 h (Figure 1b). A data reduction step was carried out in the following diagrams for a more straightforward presentation, in which only every fifth or ninth recorded data point was plotted. A Hoechst concentration of 256 ng/10^4^ (1.8 × 10^−6^ m ) cells significantly increased cell death after 36 h, as confirmed in another study.^[^ ^12^ ^]^ Proliferation was significantly repressed from 8 ng/10^4^ (57 × 10^−9^ m) cells Hoechst onwards. With a concentration of 4 ng/10^4^ cells Hoechst (28.5 x 10‐9 M), cells showed a similar proliferation rate as cells treated without Hoechst (Figure 1b,c and Video S1, Supporting Information).

To determine the minimum Hoechst concentration required to stain the cells for 5 days of long‐term LCI, we seeded 5 × 10^3^, 1 × 10^4^, and 2 × 10^4^ ARPE‐19 cells in each well of a 48‐well plate and exposed them to 4, 2, and 1 ng/10^4^ cells Hoechst. Since the molar concentration varies with different cell numbers, we calculated the respective molarity (Figure 1d). For comparing both counting methods, the cell count was normalized to one 2 h after the start of the LCI, respectively 2 h after Hoechst addition. This 2‐h time point was chosen because it takes time for all nuclei to incorporate sufficient amounts of Hoechst. For a seeding density of 5 × 10^3^/48‐well, 1 ng/10^4^ (3.5 × 10^−9^ m), and 2 ng/10^4^ (7 × 10^−9^ m), Hoechst was able to stain cells for 3 days, but not enough to last for 5 days, since the counts decreased significantly from 84 h onwards compared to 4 ng/10^4^ (14 × 10^−9^ m) concentration. In addition, automated count reveals that 14 × 10^−9^ m Hoechst had no noticeable effect on cell proliferation compared to the non‐Hoechst exposed cells. For a seeding density of 1 × 10^4^/48‐well, 1–4 ng/10^4^ Hoechst (7–28 × 10^−9^ m) could stain cells for 5 days without affecting cell proliferation. For a seeding density of 2 × 10^4^/48‐well, 4 ng/10^4^ Hoechst (57 × 10^−9^ m) slightly to a slight but insignificant reduction in cell proliferation. Taken together, we recommend a Hoechst concentration range of 7–28 × 10^−9^ m with 14 × 10^−9^ m (≈8 ng mL^−1^) as a reference value when adapting this system for its requirements, as cell proliferation is not impaired in this concentration range.

Phototoxicity is influenced by several parameters, e.g., the wavelength of the excitation light (nm), the radiation exposure (joules cm^−2^), the radiation energy (joules), and the exposure time (s). The total exposure time for time‐lapse LCI comprises the exposure time per single image, the experiment duration, and the interval length, respectively, as the number of recorded images. Since the excitation spectrum for Hoechst at 355 nm overlaps with the UV‐A wave range (315–400 nm), possible phototoxic effects should be avoided. Phototoxicity does not necessarily have to lead to cell death but can manifest itself, for example, through an altered proliferation rate.^[^ ^13^ ^]^ To test the influence of our acquisition parameters on proliferation, we seeded 5000 ARPE‐19 cells per well not stained with Hoechst. We irradiated them without or with the acquisition parameters described in the Methods section for Hoechst at 20‐min intervals for 96 h. In the beginning, the cell count was determined with the automatic cell counting method, and at the end, Hoechst was used for a precise cell count. No significant difference in cell number was found between cells irradiated for 96 h and those not irradiated (Figure 1e). Additionally, we performed a cell‐cycle analysis step at the endpoint by analyzing the frequency distribution of the Hoechst Integral (Intensity per area) from approx. 75 000 nuclei each. Again, we could not detect any change in the distribution between radiated and irradiated cells and, consequently, phototoxicity (Figure 1f).

**Figure 2:**
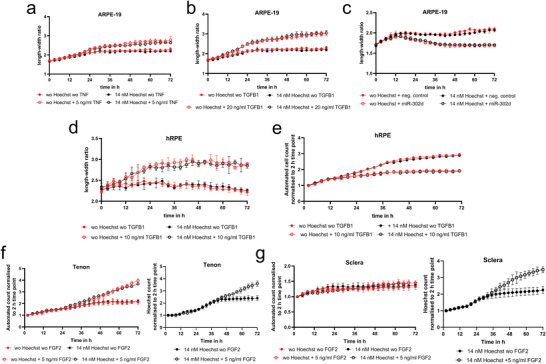
Data points of non‐Hoechst‐treated cells are highlighted in red, and Hoechst‐treated data points are shown in black. ARPE‐19 cells were treated with or without 14 × 10^−9^ m Hoechst in combination with or without a) 5 ng mL^−1^ tumor necrosis factor (TNF) or b) 20 ng mL^−1^ TGFB1 for 72 h. Both cytokines induce an epithelial‐mesenchymal transition of the cells, resulting in an increased length–width ratio. c) ARPE‐19 cells were transfected with miR‐302d, a previously reported inhibitor of EMT, or scrambled negative control and initiated EMT with TGFB1 for 72 h. Primary human RPE were treated with TGFB1 and calculated their d) average length‐width ratio and e) automated cell number for 72 h. Furthermore, we investigated the influence of the cytokine fibroblast growth factor 2 (FGF2) on proliferation in f) human tenon and g) scleral fibroblasts derived from patients who underwent trabeculectomy.

Next, we determined if 14 × 10^−9^ m Hoechst could have more subtle aversive effects and tested its influence on cell signaling pathways in three approaches. First, we treated ARPE‐19, and primary human RPE cells, with or without Hoechst, and exposed them to transforming growth factor beta 1 (TGFB1) or tumor necrosis factor alpha (TNFA) (**Figure** **2**a,b,d,e), which are both reported to induce an epithelial‐mesenchymal transition (EMT), resulting in an increased length‐width ratio.^[^ ^14^ ^]^ Second, we transfected ARPE‐19 with the microRNA miR‐302d, a previously reported inhibitor of EMT, and initiated EMT with TGFB1 (Figure 2c).^[^ ^15^ ^]^ Third, we treated primary human tenon and sclera cells with fibroblast growth factor 2 (FGF2) and measured the FGF2‐inducing effect on the proliferation rate for 72 h (Figure 2f,g).^[^ ^16^ ^]^

In all cases, we observed a cell response to respective cytokine exposure or transient miR‐302d overexpression, regardless of whether they were treated with or without 14 × 10^−9^ m Hoechst.

An exception is the comparison of automatic and Hoechst cell counting in scleral cells exposed without or with FGF2. A significant effect of FGF2 on proliferation was observed only in the Hoechst cell counting. As scleral cells are elongated fibroblasts that cannot be accurately counted automatically, this underlines the advantage of using Hoechst for cell counting.

For verification that Hoechst does not affect even more susceptible and complex processes, such as neuronal differentiation, we used Hoechst during retinoic acid (RA)‐induced neuronal differentiation of the neuroblastoma cell line SH‐SY5Y.^[^ ^17^ ^]^ Representative phase‐contrast pictures reveal no interference of Hoechst with the differentiation process (Figure S2, Supporting Information). More evidence for Hoechst's non‐impairment of cell function was impressively visible in beating rat cardiomyocytes treated with Hoechst in 2 days intervals for up to 20 days using a regular inverted fluorescence microscope (Video S2, Supporting Information).^[^ ^18^ ^]^

In the following, we present two examples of high‐throughput practical applications: cytotoxicity assays and real‐time monitoring of transduction efficiencies. For cytotoxicity assay, we performed real‐time LCI on human Tenon cells treated with different dosages of Mitomycin C (MMC) together with 14 × 10^−9^ m Hoechst and propidium iodide (PI) for 5 days.^[^ ^19^ ^]^

**Figure 3:**
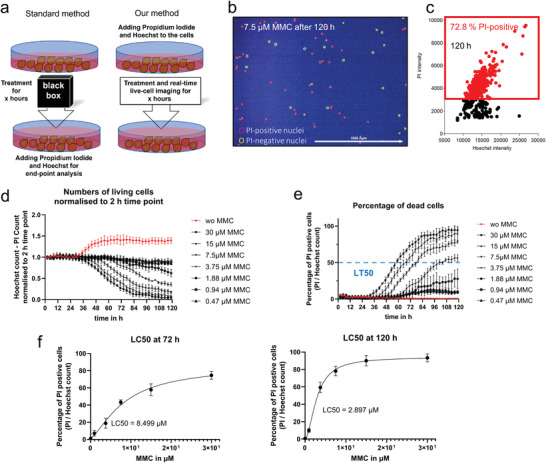
a) For cytotoxicity assays, the fluorescent dyes Hoechst and propidium iodide (PI) are added to the cells at the end of treatment. In contrast, in our method, both fluorescent dyes are added before treatment, allowing real‐time monitoring of cytotoxic effects. A total of 5 × 10^3^ human Tenon cells were treated with different dosages of Mitomycin C (MMC) and 14 × 10^−9^ m Hoechst, together with 5 ng mL^−1^ PI. LCI was performed for 120 h. b) A representative fluorescent image 120 h after adding 7.5 × 10^−6^ m MMC. Hoechst (blue) and PI (red) highlighted live and dead cells, respectively. c) A scatter plot shows Hoechst intensities versus PI intensities of tenon cells from four technical replicates 120 h after adding 7.5× 10^−6^ m MMC. 72.8% of cells were dead (PI‐positive) of the Hoechst‐stained cells. d) The changes in the numbers of alive cells (Hoechst – PI count) normalized to the 2 h time point, and e) the percentages of PI‐positive dead cells with different dosages of MMC for 5 days. MMC‐induced dosage‐dependent cell toxicity was measured in four technical replicates. In addition, the time to reach 50% lethal effect (LT_50_) of different dosages of MMC could be easily calculated. f) The lethal effect of MMC in correlation with dosages at 72 h (left) and 120 h (right). A total of 50% lethal concentrations (LC_50_) could be calculated directly in the graphs using Graph Pad Prism9.

Usually, both fluorescent dyes are added to cells for an endpoint assay, but the processes that occur beforehand remain unknown. With the assistance of Hoechst, this gap can be filled (**Figure** **3**a). Applying the Hoechst gated nuclei as a sub‐mask for the red (PI) channel and setting an intensity threshold, we can plot the number of PI‐positive nuclei or the percentage of dead cells in a scatter plot for a specific time (Figure 3b,c). In addition, the live cell count (Figure 3d) and the percentage of dead cells can be monitored for each time point, allowing us to determine the respective median lethal time (LT50) for every lethal MMC concentration (Figure 3e). Also, the median lethal concentration (LC50), an essential parameter in toxicology, is often determined for a specific time. Here, we calculated the LC50 after 72 h and 120 h (Figure 3f) as examples from our real‐time experiment data. Determining LT50 and LC50 in one run without the constraint of fixing and staining cells at specific time points is a material and time‐saving advantage. A time‐lapse video of tenon cells treated without or with 15 × 10^−6^ m MMC and the corresponding scatter plots are presented in Video S3 (Supporting Information).

**Figure 4:**
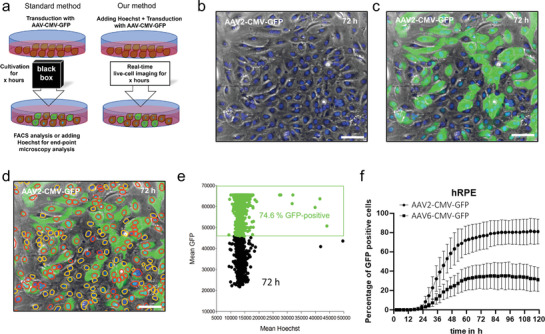
a) Typically, the transduction efficiency could be determined by adding Hoechst at the end of an experiment. However, by adding 14 × 10^−9^ m Hoechst before transduction, the transduction process in real‐time could be shown, and the transduction efficiencies could be analyzed at each time point. A total of 1 × 10^4^ hRPE cells were transfected with either AAV2‐CMV‐GFP or AAV6‐CMV‐GFP using a multiplicity of infection (MOI) of 2.5 × 10^4^ with 14 × 10^−9^ m Hoechst and performed LCI for 120 h. b) A phase‐contrast and Hoechst (blue) image 72 h after transduction of AAV2‐CMV‐GFP. c) A merged phase contrast, Hoechst, and GFP fluorescence image 72 h after transduction of AAV2‐CMV‐GFP reveal that adjacent GFP‐positive cells are difficult to distinguish. d) Hoechst staining allows the gated nuclei to be used as a sub‐mask for the green channel by setting a threshold for GFP‐positive cells. Hoechst‐stained nuclei labeled in red indicate GFP‐positive, and Hoechst‐stained nuclei labeled in yellow indicate GFP‐negative cells. e) A scatter plot shows Hoechst intensities versus GFP intensities of hRPE cells of three technical replicates 72 h after transfection of AAV2‐CMV‐GFP. A total of 74.6% of cells were GFP‐positive in all Hoechst‐stained cells. f) The transduction efficiency (the percentage of GFP‐positive cells from Hoechst‐stained cells) of AAV2 and AAV6 in hRPE cells was determined using three technical replicates at different time points for 5 days. Therefore, real‐time transduction efficiencies of different virus vectors can be calculated with Hoechst, offering a clue on choices of transfection vectors.

Furthermore, our approach can monitor transduction processes, and corresponding transfection efficiencies can be analyzed at each time point (**Figure** **4**a). We transduced hRPE cells exposed to 14 × 10^−9^ m Hoechst with either AAV2‐CMV‐GFP or AAV6‐CMV‐GFP using an Multiplicity of infection (MOI) of 2.5 × 10^4^, and LCI was performed for 120 h. The phase‐contrast image merged with the Hoechst and GFP channels reveals that adjacent GFP‐positive cells are difficult to distinguish and cannot be adequately gated in the green channel (Figure 4b,c). With the help of Hoechst, it is possible to apply the Hoechst mask as a sub‐mask for the green channel in combination with an intensity threshold to separate GFP‐positive cells from GFP‐negative cells (Figure 2d,e). The transduction efficiency can be monitored over several days, and screening for suitable serotypes for a specific cell type can be achieved. Here, we exemplary compared the AAV2 with the AAV6 serotype in human RPE cells for 5 days (Figure 2f). A time‐lapse recording of transduced cells in combination with the corresponding scatter plots is provided in Video S4 (Supporting Information).

## 3 Discussion

The presented method allows accurate cell counting using Hoechst at a minimum concentration of approximately 14 × 10^−9^ m in the medium during LCI analysis. This concentration was determined for cells cultured in 48‐well plates. When using this method for larger well formats or higher cell numbers, we recommend a maximum concentration of 1–4 ng per 10000 cells, starting with 1 ng per 10 000 cells as a guideline. Unlike automated phase‐contrast cell counting, this approach allows accurate cell counting at any time. It is suitable for all adherent cells, regardless of shape or confluence.

However, suspension cells or cells that tend to grow as multilayers are a limitation of this method because the nuclei are not likely to be in the same focal plane. In addition, this approach can be challenging for monitoring adherent cells with low cytoplasm/nucleus ratios, such as embryonic stem cells or iPS cells, where the nuclei are not far enough apart to be accurately gated.

Because Hoechst binds to AT‐rich sequences in DNA, it is thought that Hoechst inhibits both DNA replication and RNA synthesis, thereby affecting cell behavior, signaling cascades, proliferation, and cell viability. Therefore, Hoechst was considered unsuitable for real‐time LCI analysis. Numerous publications have confirmed the cytotoxicity of Hoechst. In the neuroblastoma cell line SK‐N‐SH, for example, adding 3.75 to 5 µg mL^−1^ Hoechst significantly induces cell death.^[^ ^20^ ^]^ A total of 5 µg mL^−1^ Hoechst reduces the cell viability of HL‐60 cells to 30% after 24 h.^[^ ^21^ ^]^ Erba et al. reported cytotoxic effects of 1.5 µg mL^−1^ Hoechst on L1210 cells.^[8b]^ 5 × 10^−6^ m Hoechst significantly inhibits the proliferation of human cerebral glioma cells and induces cell cycle arrest in the G2/M phase.^[^ ^22^ ^]^ Most of these reports date back to the 1980s and 1990s when fluorescence measurement devices such as FACS or fluorescence microscopes did not have the sensitivity they have today.

In our initial screening for an appropriate concentration of Hoechst, we confirmed the cytotoxicity and antiproliferative effects of Hoechst at higher concentrations. However, significant camera resolution and sensitivity advances have been made, reducing the need for high concentrations to detect a reliable signal. Since toxicity and side effects are a matter of dosage, we found that Hoechst at a concentration of 8 ng mL^−1^ or 14 × 10^−9^ m, respectively, was harmless but effective. Therefore, our recommended concentration is far below those studied by other groups reporting cytotoxic effects of Hoechst, which range from 1 × 10^−6^ to 20 × 10^−6^ m. In cell lines and primary cells, we have tested proliferation rates, or signaling cascades appear to function normally at 14 × 10^−9^ m Hoechst, similar to cells not exposed to Hoechst.

Another concern with LCI fluorescence microscopy is that the excitation light can induce phototoxicity and thus affect cell behavior and viability. Phototoxicity is a phenomenon that is influenced by many parameters.^[^ ^13^ ^]^ Primarily, the wavelength of the excitation light, the exposure time, and the light energy, which is the product of the excitation power and the illumination time, play a role. In real‐time or time‐lapse LCI, the duration of the experiment and the exposure intervals are additional parameters that determine cytotoxicity. Since we performed high‐throughput analyses, we used only a 4× objective for all experiments to capture as many cells as possible per image section. The acquisition parameters were optimized to limit possible phototoxic effects. Therefore, the camera gain was set to maximum, and the LED intensity for absorbance was set to 60% to minimize exposure times, excitation power, and, thus, incident light energy.

The advantage of using a 4× objective is that the light energy (mW cm^−2^) is distributed over a much larger area than 10× or higher magnification objectives, thus minimizing phototoxic effects. Therefore, we can recommend this method only for 4× or lower objectives because we cannot exclude phototoxic effects using high‐resolution objectives.

To investigate possible phototoxic effects, we exposed ARPE‐19 cells without adding Hoechst to the excitation wavelengths generated by our imaging parameters. Here, cells were exposed to 71 ms extinction light at 20‐min intervals for 96 h, resulting in a total exposure time of 20.45 s. Cells exposed to the corresponding extinction light showed similar proliferation compared to the non‐irradiated control group (Figure 1e,f). For comparison, another group observed a significant inhibition of proliferation by blue light starting at 4 min of irradiation.^[^ ^13^ ^]^

## 4 Conclusion

The ever‐increasing sensitivity of microscope cameras in recent years allows the use of a minimal Hoechst concentration for LCI. Our Hoechst concentration does not appear to affect cell viability or function and is far below that reported by other groups. LCI with Hoechst allows accurate cell counting before, during, and after treatment for up to 5 days. Unlike automated phase‐contrast cell counting, it allows accurate cell counting at any time. It can be used with high‐throughput automated fluorescence LCI systems or conventional inverted fluorescence microscopes.

## 5 Experimental Section

### Materials

**Table:** 

Reagent or Resource	Source	Identifier
Chemicals		
AAV2‐CMV‐eGFP	Vectorbuilder	PANEL‐AAVS01
AAV6‐CMV‐eGFP	Vectorbuilder	PANEL‐AAVS01
DMEM‐F12	Lonza	VCA‐1001
DMSO	Santa Cruz Biotechnology	sc‐358801
Dulbecco's PBS	biowest	L0615‐500
FBS Standard	PAN Biotech	P30‐3306
GlutaMAX Supplement	gibco	35050061
Hoechst	SIGMA	B2261‐25MG
Laminin	SIGMA	L2020‐1MG
Lipofectamine RNAiMAX	Invitrogen	13778‐075
MEM	SIGMA	M8042‐500ML
miR‐302d mimic	ThermoFisher Scientific	Cat # 4464066 / MC10927
miRNA negative control	ThermoFisher Scientific	Cat # 4464058
Mitomycin C	SIGMA	M4287‐5×2MG
Opti‐MEM + GlutaMAX	gibco	51985‐026
Pen Strep	gibco	15140‐122
Propidium Iodide	SIGMA	P4170‐10mg
Recombinant human FGF‐basic (FGF‐2)	Peprotech	Cat # 100–18B
Recombinant human TGFB1 (HEK293 derived)	Peprotech	Cat # 100–21
Recombinant human TNF‐*α*	Peprotech	300‐01A
Retinoic acid	SIGMA	R2625
TrypLE Express	gibco	12604‐021
Experimental Models: Cells/Cell Lines		
ARPE‐19	ATCC	CRL‐2302
Primary Human RPE Cells	This Paper	N/A
Primary Human Sclera Cells	This Paper	N/A
Primary Human Tenon Cells	This Paper	N/A
Primary rat ventricular cardiomyocytes	Wollert et al., 1996	N/A
SH‐SY5Y cells	ATCC	CRL‐2266
Software		
Gen5 V3.05	Agilent	www.agilent.com
Gen5 V3.12	Agilent	www.agilent.com
Microsoft Excel 2016	Microsoft	www.microsoft.com
Movavi Video Suite 2022	Movavi	www.movavi.com
GraphPadPrism9	GraphPad Software	www.graphpad.com
Other		
Lionheart Biotek LCI	Agilent	www.agilent.com
Microscope Zeiss Axiovert Observer Z1	Carl Zeiss	www.zeiss.com

### Cell Culture

ARPE‐19 cells were cultured in DMEM/F12 containing 10% FBS, 1× P/S, and 1× Glutamax. For LCI, ARPE‐19 cells between passages 8 and 15 were used. Tenon and scleral tissues were obtained from patients who underwent trabeculectomy. Human RPE cells were isolated from patients undergoing enucleation. The procedures were carried out according to the Declaration of Helsinki and with the approval of the Hannover Medical School Ethics Committee (Study Nr. 99931_BO_K_2021). An informed written agreement was obtained from all subjects. Tenon and sclera tissues were cultured as a tissue attachment explant in MEM Eagle containing 10% FBS, 1× P/S, and 1× Glutamax in a 12‐well.^[^ ^16^ ^]^ Approximately 1 week later, an outgrowth of TFs from the tissue could be observed. Both cell types were split at around 90% confluence, and cells between passages two and five were used for LCI. Human RPE cells were derived from patients undergoing enucleation. The enucleated eye was transferred into a 50 mL Falcon tube filled with MEM during surgery. Before preparation, the eye was placed in 70% ethanol for 30 s and transferred to a 10 cm petri dish filled with HBSS. The eye was cut in two halves, and the vitreous humor and retina were removed. The exposed RPE sheet was transferred to a 2 mL Eppendorf tube and washed twice with HBSS. Then 1 mL TrypLE Express was added to the RPE sheets and incubated for 30 min at 37° and 400 rpm on a thermal shaker. The RPE cells removed from the sheet were centrifuged at 200 rcf for 2 min, and the cell pellet was carefully resuspended in 1 mL MEM with 10% FBS, 1× P/S, and 1× Glutamax and transferred to a well of a 12‐well plate. The sheet was digested twice with TrypLE, and the RPE cells were isolated as described above. Fresh medium was added to the cells every 3 days. At ≈90% confluence, RPE cells were split in a 1:1 ratio. For LCI, RPE cells between passages one and five were used. The SH‐SY5Y neuroblastoma cell line was obtained from ATCC (CRL‐2266). The medium was DMEM/F12 (1:1) supplemented with 10% FBS, Glutamax, and 1× P/S. When cells were seeded for neuronal differentiation, 5% FBS was used, and each well of a 24‐well plate was coated with 200 µL of a 25 mg mL^−1^ Laminin–PBS solution.

Prof. T. Kempf kindly provided rat ventricular cardiomyocytes. A detailed protocol for isolation and cultivation is described here.^[^ ^18^ ^]^

### Hoechst Staining

A total of 10 mg Hoechst was dissolved in 10 mL sterile bidest and sterile‐filtered using a 33 mm diameter PES syringe filter with a pore size of 0.22 µm. The Stock solution was aliquoted and stored at −20 °C. Before LCI, the stock solution was further diluted in PBS, and the appropriate Hoechst concentration was added directly to the culture medium of the cells without additional washing steps.

### Cytokine Treatments

A total of 1 × 10^4^ ARPE‐19 cells were seeded in DMEM/F12 + 10% FBS in each well of a 24‐well plate. The next day before LCI, the media was changed to 2% FBS containing 14 × 10^−9^ m Hoechst and either 5 ng mL^−1^ TNF or 20 ng L^−1^ TGFB1. The same procedure was performed for hRPE exposed to TGFB1 but with MEM instead of DMEM/F12. One day before, 5 × 10^3^ tenon or sclera cells were seeded in MEM + 10% FBS in each well of a 48‐well. Before LCI, the media was replaced with MEM + 10% FBS containing 14 × 10^−9^ m Hoechst and 5 ng mL^−1^ FGF2.

### Retinoic Acid (RA) Treatment

SH‐SY5Y cells were differentiated to neuron‐like as previously described with minor changes.^[18b]^ One day before RA treatment, wells of a 24‐well plate were coated with 20 µg mL^−1^ laminin for 3 h. After removing the laminin solution, 2.5 × 10^4^ cells were seeded in DMEM/F12 + 5% FBS in each well of a 24‐well plate without or with 14 × 10^−9^ m Hoechst. Before LCI, none, 10 × 10^−6^ or 20 × 10^−6^ m retinoic acid was applied. Half of the medium was changed after 48 h containing 14 × 10^−9^ m Hoechst.

### microRNA Transfection and TGFB1 Exposure

One day before, 1 × 10^4^ ARPE‐19 cells were seeded in DMEM/F12 containing 10% FBS in each well of a 48‐well plate. Before transfection, the media was changed to DMEM/F12 + 2% FBS containing 14 × 10^−9^ m Hoechst and 20 ng mL^−1^ TGFB1. Cells were transfected with 5 pmol miR‐302d or 5 pmol of a scrambled negative control using Lipofectamine RNAiMax according to the manufacturer's protocol before LCI.

### Mitomycin C Treatment

One day before LCI, 5 × 10^3^ primary human tenon cells were seeded in MEM + 10% FBS in each well of a 48‐well plate. Media was changed to MEM + 10% FBS with 14 × 10^−9^ m Hoechst and 5 ng mL^−1^ Propidium iodide, and Mitomycin C in a concentration range between 30 × 10^−6^ and 0.47 × × 10^−6^ m was added before LCI.

### LCI of Rat Cardiomyocytes with a Regular Inverted Fluorescence Microscope

A total of 1 × 10^6^ primary rat cardiomyocytes were seeded in a 3 mL plating medium in a 3 cm cell culture dish.^[^ ^18^ ^]^ A prewarmed plating medium containing 14 × 10^−9^ m Hoechst was added to the cells every second day before recording. A phase‐contrast recording and the corresponding Hoechst recording were performed immediately. The Zeiss Axiovert inverted fluorescence microscope equipped with an Axiocam 503 mono camera and a CCD Sony ICX 674 sensor chip was used for this purpose. The Zen software and live image preview were used to observe beating cardiomyocytes. Short video clips were recorded with the Movavi screen recorder every second day for 20 days, and the individual video clips were edited into one video using Movavi Video Suite.

### Transduction of hRPE with AAV2/AAV6‐CMV‐GFP

Briefly, 1 × 10^4^ primary human RPE cells were seeded in MEM + 10% FBS in each well of a 48‐well plate. The next day, Media was changed to MEM + 10% FBS with 14 × 10^−9^ m Hoechst. Before LCI, RPE cells were transduced with the adenovirus‐associated particles AAV2‐CMV‐GFP or AAV6‐CMV‐GFP purchased from VectorBuilder using an MOI of 25,000.

### Live‐Cell Imaging

LCI experiments were performed using the BioTek Lionheart FX Automated Microscope with a CCD Sony ICX 285 camera Chip. The experiment setup and subsequent analysis were performed using the Gen5 image prime 3.05 software. The 48‐well culture plate was placed in the humidity chamber with CO_2_ levels set to 5% and temperature to 37 °C. Imaging was carried out in the phase‐contrast channel using the 4× PL FL phase objective and laser autofocus. In addition to the phase‐contrast images, the DAPI fluorescence filter set was used to detect Hoechst‐stained nuclei. The RFP fluorescence filter was used to detect PI‐positive nuclei, and the GFP fluorescence filter was used to detect GFP expression. Images were recorded for 72 h to 120 h at 20 or 30‐min intervals. The acquisition parameters for the phase contrast channel were set to 10 “LED,” 100 ms “Integration time,” and 4.8 “gain.” Since Hoechst is excited by near‐UV light, we used relatively high gain settings to keep the emission and exposure time as short as possible to reduce phototoxic and potential photobleaching effects. Therefore, the values were set to 6 “LED,” 71 ms “Integration time,” and 24 “gain” for the DAPI channel. For RFP, the values were set to 10 “LED,” 100 ms “integration time” and 24 “gain,.” For GFP, the values were set to 8 “LED,” 90 ms “Integration time” and 24 “gain.”

### Cell‐Cycle Analysis

For the cell cycle analysis in Figure 1e, the cells were fixed by adding 250 µL 4% PFA for 20 min in each well of a 48‐well. After washing with 500 µL PBS twice for 5 min, the nuclei of cells were stained with 250 µL Hoechst (1 µg mL^−1^ in PBS) for 30 min. After two additional washing steps with PBS, the nuclei were imaged in the live‐cell imager with the 10× PL FL phase objective. For the DAPI channel, the values were set to 5 “LED,” 65 ms “Integration time,” and 0 “gain.” A montage of 7 × 8 images with a 100 µm overlap of each well and 12 technical replicates (wells) were taken to include as many cells as possible for further analysis. Next, an image stitching image process was performed using the “Linear Blend” fusion method and a downsize image reduction step of 80%. The stitched images were further preprocessed due to a background subtraction process. Here, the “Background” was set to “Dark,” flattening was set to “Auto” with a rolling ball parameter of 888 µm, and the “Image Smoothing” strength was set to “0.” For the object counting step, the “Background” was set to “Dark,” and the “Threshold” was set to “5000.” “Split touching objects,” “Fill holes in mask,” “Analyse entire image” were checked, and under “Advanced Detection Options” a 20 µm “Rolling Ball Diameter” was used for “Background flattening.” Nuclei were finally gated by using an object size between 7 and 50 µm, and the “Integral[Tsf[Sitched[DAPI 377447]]]” was selected under “Calculated Metrics.” After nuclei count analysis, the integral (Hoechst intensity per area) of 12 technical replicas was determined from an average of 75 000 nuclei. The Hoechst integral data points were further analyzed using Graph Pad Prism 9. Here, the analysis step “Frequency Distribution” was performed by tabulating “Relative frequency (percentages)” and setting the “Bin width” to 100,000.

### LCI Analysis

For the LCI analysis, Gen5 software 3.12 was used. Phase‐contrast images were modified for the automated cell count using the “Image preprocessing” tool. Here, “Background flattening” on a “Light” background was chosen using a “Light” a “Rolling ball diameter” of 40 µm, “Priority” was set to “Fine results,” and “Imaging smoothing strength” was set to 10. The cell number was calculated using the cell count option. There, the “Threshold” was set to 2000, the background to “Light” and the options “Split touching objects” and “Fill holes in masks” and “Analyse entire Image” were selected. Under “Object selection” a “Min. object size” of 15–30 µm and a “Max. object size” of 70–200 µm was specified depending on the cell type. The number of DAPI‐positive nuclei per image was determined using the cell count option. For this purpose, the analysis tool was used, and the channel “DAPI 377, 4747” was selected under “Primary mask and Count”. The “Threshold” was set to 2000, the background to “Dark” and the options “Split touching objects” and “Fill holes in masks” were selected. In addition, under “Advanced Detection Options” a “Background flattening” with a “Rolling Ball diameter” of 30 µm, an “Image smooth strength” of 2 “Cycles of 3 × 3 average filter,” and “Evaluate background on” 5% of “lowest pixels” were selected. Under “Object selection” a “Min. object size” of 10 µm and a “Max. object size” of 70 µm were specified, and “Analyse entire image” was selected. The number of PI‐positive objects was calculated with the analysis tool for counting dead cells. Therefore, under “Primary mask and Count,” the channel “RFP 531, 593” was selected. The gating parameters previously described for DAPI were used for further analysis. The number of PI‐positive cells was divided by the number of Hoechst‐positive nuclei for each time point to determine the percentage of dead cells. The mean and standard deviation of technical replicates were calculated using GraphPadPrism 9.

### Statistical Analysis

The data was further analyzed using Microsoft Excel 2010 and GaphPadPrism 9. For a more concise presentation, only every fifth (for 72 h LCI) or ninth data point (for 120 h LCI) was displayed in Graph‐PadPrism 9. For Figures 1b,d and 2, a two‐way ANOVA test comparing Hoechst‐stained samples to the non‐stained sample for each time point was performed to reveal any significant differences using GraphPadPrism 9. A *p*‐value < 0.05 was considered statistically significant.

## Conflict of Interest

The authors declare no conflict of interest.

## Author Contributions

H.F. performed supervision, conceptualization, methodology, validation, formal analysis, and investigation, writing the original draft, and project administration. K.J. performed methodology, investigation, writing, reviewing, and editing the work. X.H. performed investigation, writing, reviewing, and editing the work. R.M. performed investigation, writing, reviewing, and editing the work. M.B. performed investigation, writing, reviewing, and editing the work. C.F. performed writing, reviewing, editing, and funding acquisition.

## Supporting information

Supporting Information

Supplemental Video 1

Supplemental Video 2

Supplemental Video 3

Supplemental Video 4

## Data Availability

The data that support the findings of this study are available from the corresponding author upon reasonable request.
