# Supplementary material for: Breaking a Dogma: High‐Throughput Live‐Cell Imaging in Real‐Time with Hoechst 33342
Source: Adv Healthc Mater. 2023 Mar 31;12(20):2300230. doi: 10.1002/adhm.202300230 (PMC11468280; doi:10.1002/adhm.202300230)
Supplement: Supplementary file 1 — Supporting Information [file ADHM-12-2300230-s005.pdf]

ADVANCED HEALTHCARE MATERIALS

Supporting Information

for *Adv. Healthcare Mater.*, DOI 10.1002/adhm.202300230

# Breaking a Dogma: High-Throughput Live-Cell Imaging in Real-Time with Hoechst 33342

*Heiko Fuchs*, Kirsten Jahn, Xiaonan Hu, Roland Meister, Maximilian Binter and Carsten Framme*

Supporting Information

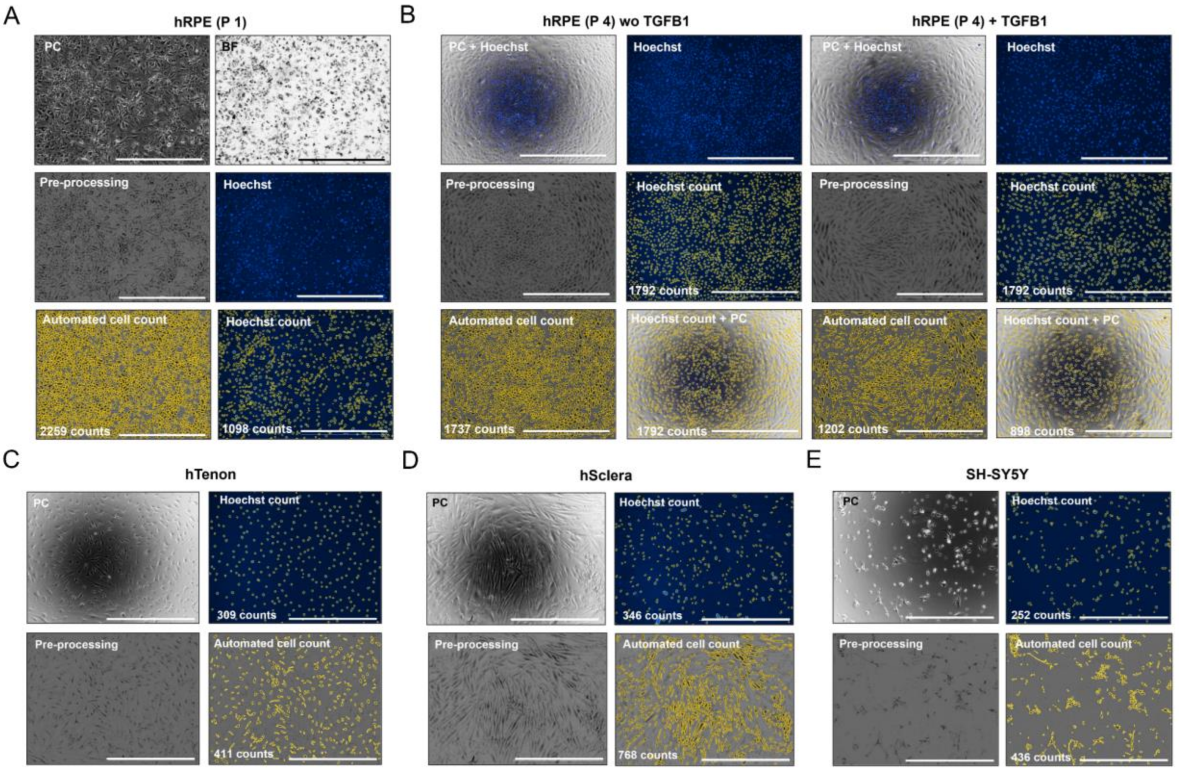


**Figure S1:** Comparison between automated cell counting and Hoechst counting on different cell types. For the automatic cell count, the phase contrast image was preprocessed, and dark objects on a bright background were subsequently gated. The blue fluorescent nuclei were gated on a dark background without preprocessing steps for the Hoechst count. The scale bar represents 1000 µm. **a)** RPE cells at passage one show intense pigmentation, as seen in the bright-field image, which is also present after the preprocessing step and prevents accurate automated cell counting. **b)** In contrast, hRPE cells derived from the same patient lose their pigment from passage 4. Compared to the Hoechst count, the automated count seems to be accurate. However, this can be negatively influenced by cell morphological changes during LCI experiments. For example, we induced EMT of hRPE with TGFB1, causing cells to change their roundish morphology and elongate. Primary human tenon **c)** and sclera **d)** are fibroblast-like cells with considerable variations in their cell length, making accurate cell gating impossible. **e)** In the neuroblastoma cell line SH-SY5Y, spontaneous or induced differentiation into cell types with different sizes and shapes can occur, resulting in inaccurate cell gating.

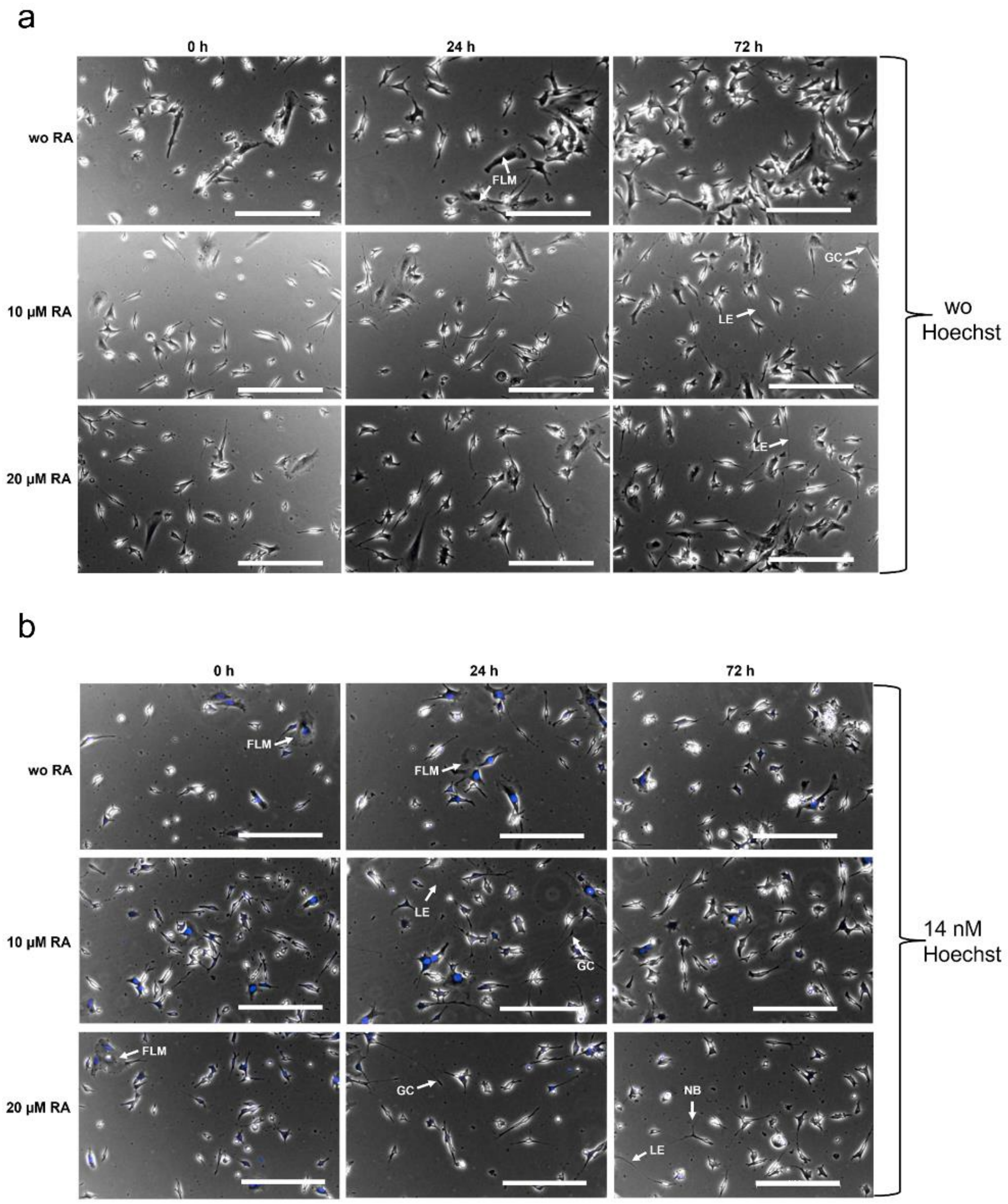


**Figure S2:** Representative phase-contrast images of the neuroblastoma cell line SH-SY5Y treated without **a)** or with 14 nM Hoechst **b)**, together without or with 10 µM or 20 µM Retinoic Acid (RA) after 0, 24, and 72 hours.
In both groups, SH-SY5Y cells developed long extensions (LE), growth cones (GC), and neurite branching (NB) in response to RA, revealing no interference of Hoechst with the differentiation process. Besides the neuronal differentiation, fibroblast-like morphologic (FLM) cells could be observed, mainly in the RA-untreated cells. The scale bar represents 250 µm.

**Video S1:** Testing different Hoechst concentrations on ARPE-19 cells
The video shows representative LCI recordings of ARPE-19 cells exposed to none, 4 ng, 32 ng, and 256 ng / 104 cells Hoechst in 20 min-intervals for 72 h. Additionally, 5 ng/ ml Propidium Iodide was added to stain dead cells.

**Video S2:** Primary rat cardiomyocytes exposed to 14 nM Hoechst every second day still show contractions *in vitro* for 20 days in culture.
The video shows a series of phase-contrast and Hoechst recordings of beating rat cardiomyocytes taken for 20 days in 2 days-intervals with an inverted fluorescence microscope. Before each recording,

a fresh, pre-warmed cardiomyocyte medium with 14 nM Hoechst was added to the cells. The replay speed was increased fourfold to shorten the video.

**Video S3:** Monitoring real-time cytotoxicity on primary human tenon cells treated without or with 15 µM MMC in combination with Hoechst and propidium iodide (PI).
The video shows time-lapse videos of human primary tenon cells treated without (top) or with 15µM Mitomycin C (bottom). 5,000 Cells were stained with 14 nM Hoechst, 5 ng/ml PI and recorded in 20-min intervals for 120 h. On the left, fluorescence videos of Hoechst-labeled tenon cells are shown. Yellow circles indicate PI-negative cells, and red circles highlight PI-positive cells. The scale bar represents 1000 µm. On the right are corresponding XY scatter plots (Hoechst intensity versus PI intensity) for each time point from 3 technical replicates. PI-negative cells are shown in black, and PI-positive cells are highlighted in red.

**Video S4:** Monitoring real-time transduction efficiencies in human primary RPE cells transduced with AAV2- or AAV6-CMV-GFP particles in combination with 14 nM Hoechst.
The following video shows time-lapse videos of human RPE cells transduced with AAV2-CMV-GFP (top) or AAV6-CMV-GFP (bottom) with an MOI of 25,000. 1 x 104 cells were stained with 14 nM Hoechst and recorded in 30-min intervals for 120 h. On the left, fluorescence videos with gated Hoechst-labeled nuclei are shown. Yellow circles indicate GFP-negative cells, and red circles highlight GFP-positive cells. The scale bar represents 500 µm. On the right, corresponding XY Scatter plots (Hoechst intensity vs. GFP intensity) are shown for each time point from 4 technical replicates. GFP- negative cells are shown in black, and GFP-positive cells are highlighted in green.
